# Rare Primary Malignant Bone Sarcomas

**DOI:** 10.3390/cancers12113092

**Published:** 2020-10-23

**Authors:** Emanuela Palmerini, Alberto Righi, Eric L. Staals

**Affiliations:** 1 Chemotherapy Unit, IRCCS Istituto Ortopedico Rizzoli, 40136 Bologna, Italy; 2 Surgical Pathology, IRCCS Istituto Ortopedico Rizzoli, 40136 Bologna, Italy; alberto.righi@ior.it; 3 Orthopaedic Surgery, IRCCS Istituto Ortopedico Rizzoli, 40136 Bologna, Italy; eric.staals@ior.it

**Keywords:** bone tumor, sarcoma, rare tumor, molecular diagnostic, chemotherapy, spindle cells sarcoma, round cells sarcoma, vascular sarcoma, CIC-DUX4, BCOR-CCNH3

## Abstract

**Simple Summary:**

Primary malignant bone tumors are infrequent cancers. More than 90% of these neoplasms are classified as osteosarcomas, Ewing sarcomas or chondrosarcomas, and their clinical presentation, diagnosis, and treatment principles are well-established. The entities described in this article, are ultra-rare varieties of bone sarcomas, and there clinical and histological characteristics are not well known. Therefore, they are very difficult to be diagnosed and there is a lot of uncertainty on their treatment. Because of their rarity, it is also extremely difficult to perform clinical research on these cancers. This article creates more awareness of these very rare bone tumors. It explains how to recognize and diagnose each entity and it summarizes the medical scientific literature that is available on these cancers. Increasing awareness and clinical research for these cancers are key elements to improve the prognosis for patients with these diseases in the long term.

**Abstract:**

Rare primary malignant bone sarcomas (RPMBS), other than osteosarcoma, chondrosarcoma, chordoma, and Ewing sarcoma, account for about 5–10% of primary bone tumors and represent a major diagnostic challenge. These tumors include spindle cell and round cell sarcoma entities, hemangiopericytoma-like and vascular tumors. Additionally, several histotypes, traditionally described in the soft tissues, such as myxofibrosarcoma, synovial sarcoma, and malignant peripheral nerve sheath tumor of bone, have been reported in patients with primary bone tumors. While wide surgical resection is the mainstay of local treatment, systemic therapy of these rare entities is controversial. Patients with undifferentiated spindle cell or pleomorphic high-grade tumors of bone, are usually treated with osteosarcoma-like chemotherapy, while patients with round cell and undifferentiated round cell tumors (URCTs), may respond to sarcoma treatment regimens for Ewing sarcoma patients. Studies on analogies and differences among these ultra-rare tumors have seldom been reported. This review describes relevance, clinical aspects, diagnostic procedures, staging, treatment recommendations, and current research in this composite tumor group.

## 1. Introduction

**Figure 1:**
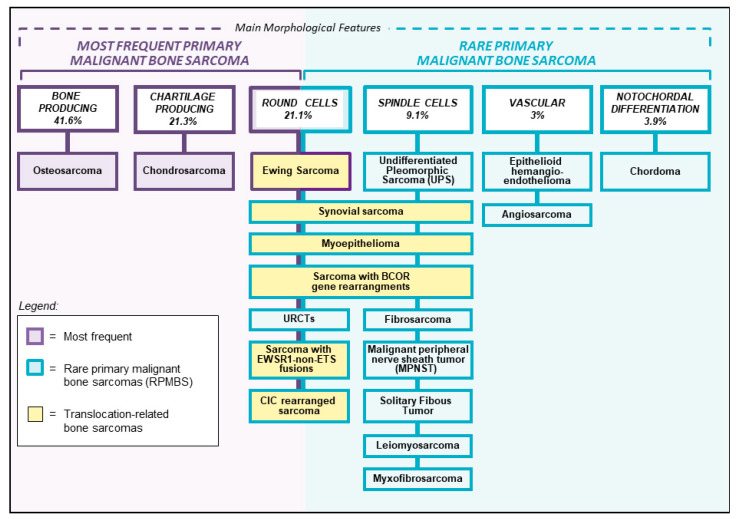
Diagnostic classification of rare primary malignant bone sarcoma. URCTs: undifferentiated round cells tumors; SFT: solitary fibrous tumor.

Primary bone sarcomas account for approximately 0.2% of all malignant tumors [1]. Osteosarcoma, Ewing sarcoma, and chondrosarcoma are the most frequent entities and represent 90–95% of all primary bone sarcomas [1]. Their clinical, radiographic, and histological characteristics are well known and there is general consensus on their management according to national and international treatment protocols in referral centers for bone sarcomas. Rare primary malignant bone sarcomas (RPMBS), that cannot be classified as osteosarcoma, Ewing sarcoma, or chondrosarcoma, represent diagnostic challenges. These tumors can have a wide variety of clinical and radiographical presentations and their diagnostic work-up include specific histopathology, immunohistochemistry, and molecular genetics analyses. RPMBS can be classified according to their histopathological characteristics into spindle cell sarcomas, round-cell sarcomas, vascular neoplasms, and rare entities that usually arise in the soft tissues like synovial sarcoma, myxofibrosarcoma, and malignant peripheral nerve sheath tumor (Figure 1). Due to their extreme rarity, there are very limited studies available on these specific entities, for which no standard treatment protocols have been defined. Therefore, their treatment strategy is usually determined on an individual basis. This review describes these rare entities of primary bone sarcomas and presents an overview of the available literature.

## 2. Relevance

This study summarizes the current situation and future potential research developments for RPMBS. It underlines the complexity of the most recent version of the World Health Organization (WHO) classification for bone tumors and the diagnostic challenges for these histotypes. Due to the rarity of these tumors, no specific prospective clinical trial is currently available for patients with RPMBS, and there is general lack of knowledge on this topic. Furthermore, this review represents an open invitation to those involved in the field of sarcoma research, to consider these entities for specific clinical trials or basket studies.

## 3. Clinically Relevant Aspects

There is no consensus on specific treatment of RPMBS. Historic data on malignant fibrous histiocytoma (MFH), a diagnosis that no longer exists but overlaps in part with entities that are currently included in the group of RPMBS, showed poor survival (21%) for patients with localized disease treated with surgery or radiotherapy alone [2]. As multimodality treatments offer the best survival for patients with the most frequent primary bone sarcomas (osteosarcoma and Ewing sarcoma), this approach is thought to improve also the oncologic outcome for patients with RPMBS.

## 4. Diagnostic Procedures

Over the past two decades, an increasing amount of genetic data on bone tumors has become available. Additionally, newly developed diagnostic tools, like new immunohistochemical markers and molecular analyses, have contributed to a deeper knowledge of bone neoplasms. These recent advances have led to a new terminology and more detailed classification.

In consideration of these new diagnostic procedures, the routine decalcification of bone tumor specimens, which is essential for proper morphology that still remains the cornerstone of the diagnosis, causes a severe limitation when implementing routine molecular testing for bone tumors [3].

Spindle cell and round cell sarcoma entities, and vascular tumors was better delineated with the advent of next-generation sequencing, and their workup using immunohistochemistry and molecular testing in daily practice is reported.

### 4.1. Spindle Cell Sarcomas

In general, histopathological diagnosis of soft tissue spindle cell sarcomas is one of the most challenging areas of surgical pathology, even more arduous is the accurate diagnosis of a spindle cell sarcoma arising in an exceptional location. Such is the case of spindle cell sarcomas originating in bone, where it is now very well recognized that a wide variety of spindle cell sarcomas most often arising in soft tissues may actually present as primary neoplasms [4,5,6,7,8].

The use of outdated labels, such as hemangiopericytoma and malignant fibrous histiocytoma, has obstructed the identification of specific entities within this group of primary spindle cell malignant neoplasm of bone. Adequate sampling and large panels of immunohistochemistry are necessary for their accurate diagnosis.

**Figure 2:**
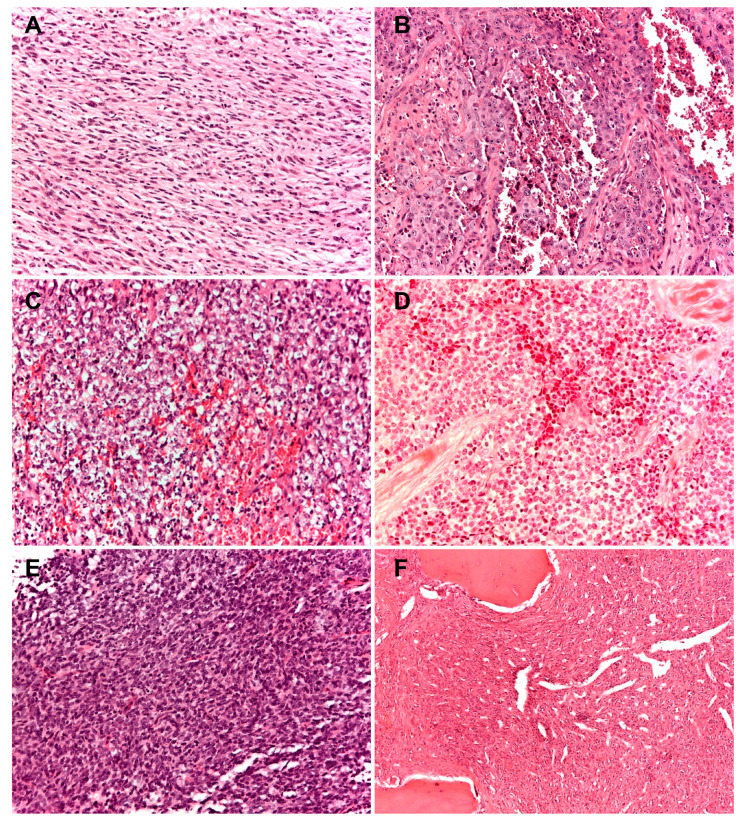
Histopathologic presentation of six cases of rare primary malignant bone sarcomas (**A**) spindle cell sarcoma of bone: tumor cells are typically organized in a fascicular or herringbone pattern; (**B**) angiosarcoma of bone: tumor is composed of a markedly atypical, predominantly solid epithelioid cell proliferation, featuring abundant eosinophilic cytoplasm, often harboring macronucleolated vescicular nuclei; (**C**–**E**) round cell sarcomas; (**C**) CIC-DUX4; (**D**) undifferentiated round cells sarcoma; (**E**) BCOR-CCNB3; (**F**) solitary fibrous tumor of bone with a classic hemangiopericytomatous pattern. (100× of magnification; haematoxylin and eosin staining).

Historically, the term “fibrosarcoma of bone” has been applied to primary malignant spindle cell neoplasms of bone in which the tumor cells are typically organized in a fascicular or herringbone pattern [5,6,7,8] (Figure 2). However, a variety of primary bone tumors occupying other specific diagnostic categories may also show this histological pattern; therefore, there are no properties distinctive of or specific for fibrosarcoma of bone [6,7,8,9]. This term is uncommonly used as a specific diagnostic category today, particularly due to the advent of ancillary techniques and evolving classification schemes [4,10]. The diagnosis of fibrosarcoma as well as of undifferentiated pleomorphic sarcoma are one of exclusion because of lack of morphological, immunohistochemical, and genetic features suggesting an alternative diagnosis.

An important differential diagnosis of primary fibrosarcoma of bone is represented by sclerosing epithelioid fibrosarcoma, that is an infrequent, molecularly defined subtype of sarcoma with fibroblastic differentiation, characterized by cords and nests of monomorphic epithelioid neoplastic cells immersed in a conspicuous sclerotic extracellular matrix. Sclerosing epithelioid fibrosarcoma can occur as either a primary or a metastatic bone lesion [11,12]. In this context, the presence of striking epithelioid morphology combined with MUC4 immunopositivity or demonstration of a fusion between *FUS* or *EWSR1* and one of the CREB3L genes allows accurate classification.

Leiomyosarcoma (LMS) of bone is a primary malignant neoplasm of bone showing smooth muscle differentiation. The lesions resemble LMSs from other locations, with cells arranged in long, intersecting fascicles, growing in an infiltrative pattern [13,14,15]. The tumors are associated with variable degrees of necrosis, nuclear pleomorphism, and mitotic activity [14,15]. Immunohistochemically, smooth muscle differentiation is demonstrated by diffuse staining with desmin and/or h-caldesmon, as well as smooth muscle. Positivity for ER or PR in female patients strongly suggests a primary uterine origin [13,16]. The distinction of LMS from low-grade central osteosarcoma or conventional high-grade fibroblastic osteosarcoma can pose difficulties. They can exhibit immunophenotypic overlap with LMS, as both tumors can express desmin. Furthermore, osteoid can be easily overlooked as it is only focally expressed in fibroblastic osteosarcomas. Careful evaluation of imaging, extensive sampling, and ruling out *MDM2* amplification by in situ hybridization techniques or surrogate immunohistochemistry, are mandatory steps in the diagnostic approach of a LMS arising as a bone primary [4].

Malignant peripheral nerve sheath tumor (MPNST) originating as a primary bone neoplasm is exceedingly rare and most often associated with the NF-1 syndrome. Classically, MPNST is most often composed of highly atypical, mitotically active, spindle cell proliferation exhibiting varied cellularity and often a distinctive perivascular aggregation of neoplastic cells [17,18,19]. Inactivation of polycomb repressive complex 2 subunit EED or SUZ12 in a majority of MPNSTs leads to loss of tri-methylation at the 27th lysine residue of the histone H3 protein, which can be detected by loss of immunoreactivity for the H3K27me3 antibody, a highly specific biomarker of MPNSTs [18].

Synovial sarcoma (SS) is a relatively prevalent spindle cell mesenchymal malignancy characterized by epithelial differentiation. SS arising as bone primary has been under-recognized until recently, partially due to the widespread use of the currently abandoned unspecific label of hemangiopericytoma [4,20,21]. Three main morphologic variants of SS exist: spindle cell monophasic, biphasic, and undifferentiated [4].

SS exhibits variable reactivity for immunohistochemical epithelial markers such as Epithelial Membrane Antigen (EMA) and cytokeratins. SS is characterized by the presence of a reciprocal t(X;18) translocation that fuses the *SYT* on chromosome 18 with *SSX1*, *SSX2*, and rarely *SSX4* on chromosome X. This translocation is pathognomonic of SS, thus it plays an important role in its diagnosis.

SS may sometimes exhibit bland morphology, such instances require differentiating it from a solitary fibrous tumor (SFT). Expression of epithelial markers, the presence of *SYT* rearrangement by FISH, and the negativity for STAT6 in the former, are all valuable diagnostic findings [8,10].

SFT is a ubiquitous fibroblastic neoplasm showing a distinctive vasculature characterized by the presence of prominent thin-walled, branching, staghorn-like vessels in a so-called hemangiopericytomatous pattern.

SFT characteristically expresses CD34 and STAT6. Diffuse and strong nuclear immunohistochemical expression of the latest is a sensitive and specific surrogate for the presence of pathognomonic *NAB2-STAT6* fusion and constitutes a very valuable immunohistochemical antibody for the diagnosis of SFT.

When presented with a SFT of bone it is mandatory to rule out an osseous metastasis from a malignant or a meningeal SFT, both exhibiting a striking tendency to spread to the bone and soft tissues [9].

Primary myoepithelial tumors of bone are very rare. Microscopically, they do not differ from myoepithelial neoplasms of the salivary glands [22,23]. Most cases are composed of spindle to epithelioid cells organized in cords, strands, or trabeculae embedded in a myxoid or myxochondroid extracellular matrix.

The presence of high-grade nuclei remains the only unequivocal histological predictor of aggressiveness [22,23]. By immunohistochemistry, up to 90% of cases express cytokeratins and S100 protein. EMA and glial fibrillary acidic protein (GFAP) can be seen in two-thirds and 50% of cases, respectively [22,23].

Approximately 50% of myoepithelial neoplasms harbor *EWSR1* fusions with a variety of partner genes (*PBX1*, *PBX3*, *ZNF44*, *POU5F1*, and *ATF1*) [24]. *FUS* has been shown to be alternatively present as the N-terminal genetic partner in oncogenic fusions in a small subset of myoepithelial tumors lacking *EWSR1* rearrangements [24].

Undifferentiated pleomorphic sarcoma (UPS) is the current designation for the now abandoned label MFH [25] and is a pleomorphic malignant neoplasm of bone with no identifiable line of differentiation. The tumor is diffusely composed of spindle-shaped and epithelioid or polygonal cells with marked pleomorphism arranged in a haphazard, storiform, and fascicular growth pattern and with numerous typical and atypical mitotic figures. Importantly, the tumor lacks any evidence of malignant osteoid or cartilage, thus necessitating thorough sampling in order to rule out osteosarcoma and dedifferentiated chondrosarcoma. A thorough immunohistochemical panel, seeking for an overlooked histologic lineage, complements the microscopic assessment of all pleomorphic neoplasms of bone. [25] The panel should include at least AE1/E3, EMA, S100 protein, smooth muscle actin, h-caldesmon, and desmin. Additional immunohistochemical or molecular auxiliary tools may be added depending on the clinicopathological characteristic of the neoplasm. Specifically, H3F3A immunohistochemistry is useful when a malignant transformation of giant cell tumor of bone is suspected [26], *IDH* mutational analysis can be performed when dealing with a doubtful high grade component of dedifferentiated chondrosarcoma [27]. Therefore, it is mandatory to integrate the clinical context before rendering a diagnosis of UPS.

Myxofibrosarcoma (MFS) is a well-defined and relatively common spindle cell sarcoma in the soft tissues, whereas in bone it has been reported only occasionally [10,28]. MFS is characterized by a nodular growth of pleomorphic cells embedded in a variable myxoid stroma, representing at least 20% of tumor surface. It exhibits a rich vasculature with characteristic curvilinear capillaries. MFS is a tumor with no specific line of differentiation that in instances can be challenging to differentiate from a UPS, since myxoid change can also occur in the latter.

### 4.2. Round Cell Sarcomas

Previously designated Ewing-like tumors, reflecting their morphological resemblance to Ewing sarcomas, but genotypically devoid of the canonical genetic fusions involving members of the ETS family of transcription factors, that are characteristic of Ewing sarcomas (Figure 2).

Clinicopathologic and molecular evidence [29] has led to the discarding the all-embracing and confusing terminology of “Ewing-like tumors”. The latest “WHO classification of soft tissue and bone tumor” classifies the undifferentiated small round cell sarcomas of bone and soft tissue into three diagnostic categories: round cell sarcomas with EWSR1non-ETS fusions, capicua transcriptional repressor (CIC)-rearranged sarcomas, and sarcoma with Bcl6 corepressor (BCOR) genetic alterations [30].

Despite significant morphologic overlap, most of the above entities can exhibit morphologic features predictive of their underlying molecular alterations. NFATC2 sarcoma may exhibit epithelioid features, and PATZ1 sarcomas often display a sclerotic background. BCOR sarcomas often contain a population of bland, short, spindle cells. CIC sarcomas can be predominantly epithelioid and may exhibit focal pleomorphism.

The differential diagnosis for round cell sarcomas of bone is rather broad, and includes alveolar rhabdomyosarcoma, poorly differentiated synovial sarcoma, small cell osteosarcoma, and mesenchymal chondrosarcoma. The substantial morphologic overlap between Ewing sarcoma and other undifferentiated small round cell sarcomas makes molecular typing mandatory for their unequivocal classification. In particular, the identification of the fusion transcript remains the gold standard. Conversely, the morphological features associated with a peculiar immunoprofile (variable CD99 staining with frequent WT1 and ETV4 positivity in CIC-rearranged sarcomas, and immunoexpression of BCOR, SATB2, and cyclin D1 associated with a positivity of CD99 in approximately 50% of cases) allow the pathologist to distinguish these small round cell sarcomas of bone. In selected cases with a doubtful immunohistochemical results, molecular confirmation of BCOR genetic abnormality or CIC gene rearrangement typing should be necessary to classify these neoplasms with certainty [31,32,33].

Approximately 11% of round cell sarcomas remain unclassified by current gene fusion panels and these are considered unclassified round cell sarcomas [34]. The use of more sensitive molecular techniques, such as RNA-sequencing, in the diagnostic workup of these neoplasms is likely to keep reducing the proportion of neoplasms that remains unclassified [29,34].

### 4.3. Vascular Tumors

The new classification of vascular tumors of bone proposed in the latest WHO classification of soft tissue and bone tumor is based on the recent discovery of novel and pathognomonic translocations in different vascular entities [35,36]. The refinement in the diagnosis of vascular neoplasms of bone also includes the incorporation of tumor types previously reported only in soft tissues, such as pseudomyogenic hemangioendothelioma and retiform hemangioendothelioma [37,38,39,40,41,42,43]. The term hemangioendothelioma alone is now obsolete and instead it refers to a broad spectrum of different vascular neoplasms.

Chromosome translocations involving the FOS gene have been identified as a genetic hallmark of epithelioid hemangioma, whilst rearrangements involving FOSB have been detected in pseudomyogenic hemangioendothelioma and in a subset of epithelioid hemangioma, defined as cellular/atypical variant. Thus, these alterations represent important diagnostic markers that can help to single out these two entities from other vascular tumors [38,42,44]. Moreover, two novel recurrent gene fusions (WWTR1–CAMTA1 and YAP1-TFE3 gene fusions) have been identified in epithelioid hemangioendothelioma [37,43,45]. So far, these genetic aberrations have never been reported for any of the morphologic mimics of epithelioid hemangioendothelioma, thus representing an additional diagnostic tool. Interestingly, these fusions lead to the mutually exclusive nuclear accumulation of CAMTA1 or TFE3, making IHC a reliable read out for both variants of epithelioid hemangioendothelioma [37,43,45]. The integration of morphological, immunohistochemical, and molecular features allows a better stratification of primary vascular tumors of bone with significant prognostic and therapeutic implications [46].

## 5. Staging

All new cases of RPMBS should be formally discussed in a multidisciplinary team at a bone sarcoma reference center with a radiologist, pathologist, surgeon, radiation oncologist, and medical oncologist with experience in the treatment of bone tumors.

General staging should assess local tumor extension and the presence of metastatic disease. Chest CT scan is mandatory and bone scintigraphy or PET-FDG should be performed [47,48,49]. Whole-body MRI is increasingly used for staging (including detection of ‘skip’ bone lesions) for most common bone sarcomas [50] and could, thus, also represent a useful tool for RPMBS. Additional appropriate imaging studies and biopsies are required for all suspicious areas.

## 6. Treatment Recommendations

As all malignant primary bone sarcomas, the cornerstone of treatment is wide surgical resection [51]. Use of chemotherapy and/or radiotherapy has to be decided on an individual basis by a multidisciplinary team of sarcoma experts, considering tumor (histotype, site, size, localization, and stage) and patient characteristics (age, comorbidities, and symptoms).

### 6.1. Spindle Cell Sarcomas

Historically most spindle cell RPMBS were diagnosed as MFH [52], and treated according to osteosarcoma-like chemotherapy regimens (adriamycin, cisplatin, ifosfamide, and methotrexate), with a lower rate of good histologic response to induction chemotherapy but survival comparable to that of high grade osteosarcoma. As reported, MFH patients were generally older than patients with high grade osteosarcoma.

Recently, the results of a subgroup of patients from the EUROpean Bone over 40 Sarcoma Study (EURO-B.O.S.S.) was presented [53]. The EURO-B.O.S.S. study was a prospective non-controlled trial including patients between 41 and 65 years of age with bone sarcomas of various histotypes, treated with wide surgical removal of the tumor with the addition of a systemic treatment based on the antineoplastic drugs active against osteosarcoma (adiamycin, cisplatin, ifosfamide, methotrexate)), either as adjuvant or neoadjuvant [54]. Patients with a diagnosis other than high grade osteosarcoma or dedifferentiated chondrosarcoma were included in this subgroup analysis presented by Reichard et al. [53]. Diagnoses were as follows: 88 UPS, 20 leiomyosarcomas, 3 fibrosarcomas, and 2 angiosarcomas. The study concluded that multiagent chemotherapy (doxorubicin 60 gr/m^2^, cisplatin 90 mg/m^2^, ifosfamide 2.5 gr/m^2^, and methotrexate 8 gr/m^2^ in case of poor response to induction chemotherapy) was feasible in this patient population. Outcome was comparable to that of patients with high-grade osteosarcoma, with favorable survival associated with extremity site and complete surgical remission after neoadjuvant chemotherapy.

Due to extended use of molecular diagnostics, the diagnosis of primary bone synovial sarcoma, *SYT-SSX1* and *SYT*-SSX2, in t(X;18)(p11.*2*;q11.*2*), positive in most of the cases, has become more frequent over recent years [55]. A retrospective series on 25 patients with bone synovial sarcoma showed a 5-year overall survival of 66% and a 5-year event free survival of 38%, after surgery. Chemotherapy (doxorubicin, methotrexate, cisplatin, and ifosfamide in seven patients; ifosfamide monotherapy in one patient), and radiotherapy were used in 7/25 and 3/25 of the cases [55].

Extrapleural SFT is usually more aggressive than the pleural form, and might occur in the mediastinum, retroperitoneum, pelvis, meninges, and soft tissues [56]. Although SFT is usually considered as a clinically indolent neoplasm, the prognosis is substantially unpredictable and only partially related to morphological feature [56]. Primary SFT arising in bone are extremely rare and rarely metastasize. Unlike for SFT in the soft tissue, no dedifferentiated SFT are described in bone and there is no consensus on the definition of malignant SFT. Case reports of humerus [57,58] and spine [59] SFT, showed occurrence of long-term relapses after surgery [57,58]. These data indicate the need for long-term follow-up (>5 years) in case of bone SFT diagnosis and metastasectomy is recommended whenever possible.

### 6.2. Round Cell Sarcomas

Antonescu et al. [60] reported on 115 patients CIC-positive tumors (only 3% of the cases overall presenting in bone). Survival analysis on 52 of these cases was performed. Twenty-two received neoadjuvant chemotherapy using a similar regimen as for Ewing sarcoma, while 29 patients had an initial surgical resection with curative intent, followed by adjuvant chemotherapy in 22 patients and radiation in two cases. Interestingly, patients treated with neoadjuvant chemotherapy (*n* = 22) had a poor pathologic response in about 70% of the cases, and showed an inferior survival compared to patients managed by surgery first (*n* = 29) (*p* = 0.025). However, patients selected for neoadjuvant therapy had a larger tumor size (*p* < 0.0001) compared to patients who were managed by surgery first, which may have caused an important selection bias.

A retrospective international series reporting on treatment and outcome of 105 patients with non-Ewing round cell tumors from 14 centers demonstrated clinical differences between CIC-DUX4, BCOR-CCNB3, and URCS [61]. While most of the CIC-DUX4 tumors were located in the soft tissues, >95% of BCOR-CCNB3 arose in the bone. The median age at presentation was 30 years for CIC-DUX4, 15 years for BCOR-CCNB3, and 41 years in case of URCS. Additionally, CID-DUX4 was frequently associated with metastases at diagnosis. The local treatment was surgery in 53%, patients, surgery + radiotherapy in 27%, and radiotherapy in 13%, whereas 7% patients did not undergo local treatment. Chemotherapy was given to 85% patients: Ewing sarcoma regimen in 66%, doxorubicin/ifosfamide (Doxo/IFO) in 19%, osteosarcoma regimen in 5%. With a median follow-up of 44 months (95%CI:35–54), the 3-year overall survival rate was 95% (95% CI 68–99) in the *BCOR-CCNB3* group, 34% (95% CI 20–49) in *CIC-DUX4* (51% vs. 15% in localized and metastatic cases), and 76% in URCS (95% CI 51–89; *p* = 0.0001) [61].

### 6.3. Vascular Tumors

Malignant vascular tumors of bone are very rare and account for less than 1% of primary malignant bone tumors [46]. This group of tumors is characterized by the frequent presence of multiple synchronous lesions and includes different histopathologic entities with various biological behaviors.

The treatment strategy for epithelioid hemangioendothelioma (EHE) is controversial and includes watch-and-wait approach, surgery, radiotherapy and chemotherapy. There is evidence to support the use of chemotherapeutics and targeted therapies specifically focusing on anti-angiogenesis [62].

Boriani et al. [63] presented a cohort of 81 patients with vascular tumor of the spine including benign and malignant entities. Of the seven epithelioid hemangioendotheliomas, four were treated by en bloc resection without any other adjuvant treatments; patients presented with multiple bone and soft tissue lesions, survival patterns were very different, and successful treatment with intralesional resection with radiotherapy in some cases

Bone angiosarcomas tend to affect long tubular bones of the extremities and the axial skeleton, mainly the spine. The bones of the lower limb, particularly the femur and the tibia, are most commonly involved, followed by the pelvis, vertebral column, and the bones of the upper limbs [64].

Data on treatment and survival of bone angiosarcoma are scars, often presented together with soft tissue lesions [65] and EHE [63] or as case reports [66,67].

A retrospective study on angiosarcoma of bone by the European Muscoloskeletal Oncology Society reported on 80 patients with bone angiosarcoma treated at nine European centers. This study showed that a surgical complete remission (SCR) status was pivotal in localized patients (5-year OS 45% for SCR, 17% no SCR, *p* = 0.03) [68]. Five-year OS was significantly influenced by age and site of the tumor. After multivariate analysis, the addition of radiotherapy to surgery significantly influenced the disease-free survival (DFS) rate, whereas the use of chemotherapy lost the significance demonstrated by the univariate analysis [68].

## 7. Current Research

Due to the rarity of RPMBS, feasibility of specific prospective clinical trials in each entity is limited. Data may be extracted from basket studies or observational registries.

The improvement in molecular diagnostic of some of RPMBS identified pathogenetic translocations that might represent potential therapeutic targets.

A preclinical study on CIC-DUX4-expressing cells demonstrated a sensitive to CDK2 inhibitors, dinaciclib and SNS-032, highlighting a paradigm of functional diversification of transcriptional repertoires controlled by a genetically aberrant transcriptional regulator, with therapeutic implications [69].

Additionally, personalized xenografts developed in mice from patients’ tumor tissues could aid in the process of interpreting genomic analyses, identifying actionable leads, and relating these to the drug space. Exome sequencing and patient-derived xenografts, so-called Avatar mouse models, were developed for personalizing cancer treatment in the clinic in real time [70].

Bone sarcoma patient-derived xenografts (PDX) were successfully established from primary cell cultures of tumor samples from patients with bone tumors.

These preclinical models are faithful and stable and represent a useful tool for molecular and therapeutic investigations [71,72].

Additionally, results obtained from prospective clinical trials for soft tissue specific histotypes, could be applied for related primary bone lesions. For example, successful treatment of visceral and somatic dedifferentiated and malignant SFT was demonstrated [73] with pazopanib.

A TKI approach might be proposed for several specific bone tumors such as vascular tumors, malignant SFT of bone, and synovial sarcoma. This idea is strengthened by the fact that recently several studies showed activity of regoraferib [74,75] and other tyrosin kinase inhibitor [74,75,76,77,78,79] in patients with relapsed high-grade osteosarcoma.

## 8. Conclusions

With a lack of specific clinical trials available for these tumors, a management decision should be made in a multidisciplinary setting in tertiary referral centers, for each individual patient.

There is a need for centralized data recording, ideally in an observational or basket study, in order to obtain scientifically relevant information on these rare entities and define specific treatment pathways.

Genetic sequencing and PDXs model might provide useful information to guide a personalized approach of patients with ultra-rare bone tumors.
